# Biocompatibility Study of Curcumin-Loaded Pluronic F127 Nanoformulation (NanoCUR) against the Embryonic Development of Zebrafish (*Danio rerio*)

**DOI:** 10.3390/molecules27144493

**Published:** 2022-07-14

**Authors:** Siti Nur Sharmila Abdullah, Kalai Arasu Subramaniam, Zahir Haizat Muhamad Zamani, Seri Narti Edayu Sarchio, Faizah Md Yasin, Suhaili Shamsi

**Affiliations:** 1 Laboratory of Animal Biochemistry and Biotechnology, Department of Biochemistry, Faculty of Biotechnology and Biomolecular Sciences, Universiti Putra Malaysia, Serdang 43400, Malaysia; sitinursharmila1511@gmail.com (S.N.S.A.); kalai668@yahoo.com (K.A.S.); zhaizat90@gmail.com (Z.H.M.Z.); 2 Department of Biomedical Sciences, Faculty of Medicine and Health Sciences, Universiti Putra Malaysia, Serdang 43400, Malaysia; serinarti@upm.edu.my; 3 Department of Chemical and Environmental Engineering, Faculty of Engineering, Universiti Putra Malaysia, Serdang 43400, Malaysia; fmy@upm.edu.my; 4 Institute of Nanoscience and Nanotechnology (ION2), Faculty of Engineering, Universiti Putra Malaysia, Serdang 43400, Malaysia

**Keywords:** curcumin, Pluronic, NanoCUR, toxicity, zebrafish embryo

## Abstract

Curcumin (CUR) has been studied for its biomedical applications due to its active biological properties. However, CUR has limitations such as poor solubility, low bioavailability, and rapid degradation. Thus, CUR was nanoformulated with the application of polymeric micelle. Previous studies of CUR-loaded Pluronic F127 nanoformulation (NanoCUR) were generally prioritized toward cancer cells and its therapeutic values. There are reports that emphasize the toxicity of CUR, but reports on the toxicity of NanoCUR on embryonic developmental stages is still scarce. The present study aims to investigate the toxicity effects of NanoCUR on the embryonic development of zebrafish (*Danio rerio*). NanoCUR was synthesized via thin film hydration method and then characterized using DLS, UV-Vis, FTIR, FESEM, and XRD. The toxicity assessment of NanoCUR was conducted using zebrafish embryos, in comparison to native CUR, as well as Pluronic F127 (PF) as the controls, and ROS assay was further carried out. It was revealed that NanoCUR showed an improved toxicity profile compared to native CUR. NanoCUR displayed a delayed toxicity response and showed a concentration- and time-dependent toxicity response. NanoCUR was also observed to generate a significantly low reactive oxygen species (ROS) compared to native CUR in ROS assay. Overall, the results obtained highlight the potential of NanoCUR to be developed in clinical settings due to its improved toxicity profile compared to CUR.

## 1. Introduction

Diferuloyl methane, or its general name, curcumin (CUR), is a compound which is the major constituent of turmeric, with a scientific name of *Curcuma longa*. CUR has been used by humans for a long time. Apart from being processed in food such as spices, CUR was also well-known for its medicinal use, mostly in India and China, as traditional Ayurvedic medicine. In India, CUR powder is believed to be the reason for the healing of diabetic wound, sinusitis, cough, swelling, and several other ailments [1]. CUR that appears as yellow or golden in color possesses hydrophobic properties, which makes it insoluble in water of about 0.03 µM. In addition, CUR has been shown to be soluble in solvents at alkaline pH, chloroform, and ethanol [2]. Previous studies have reported that CUR has myriad pharmaceutical properties, such as anti-inflammatory, antioxidant, anti-bacterial [3], anti-cancer [4], anti-mutagenic, and anti-cholesterol activities [5,6].

Despite its vast advantages, CUR has some disadvantages. CUR is known for its low bioavailability because of its poor aqueous solubility [7]. Other than that, CUR has also been shown to metabolize rapidly, degrade easily at pH above neutral (pH ≥ 7), and has a low absorption rate in the gastrointestinal tract, thus making the application of CUR for medical purposes limited [8,9]. Many CUR metabolisms in in vivo studies involving healthy human patients and rats show almost negligent amounts of CUR left in the blood plasma 1 h following the oral consumption of high doses of CUR [10,11]. CUR had already been metabolized before it was able to reach and be absorbed into the blood stream. It shows that CUR has very low bioavailability, and in order to overcome the disadvantages of CUR, an effective and efficient method should be designed so that the potential of CUR as medicine will not be overwhelmed by its several downsides. One of the methods that gives promising success is the formation of a drug delivery nanoparticle system for CUR. Adhesion of CUR to agents that act as a drug carrier for CUR may increase the solubility of CUR and shows great positive results.

One of the ideas to broaden the range of CUR’s potential as a medicine, which is currently being studied extensively, is the formulation of CUR into nanoparticles with surfactant and polymers, such as Pluronic F127 (PF), to form NanoCUR [12,13]. Encapsulation of CUR inside the micellar PF has increased the survival of CUR in the gastrointestinal tract in terms of bioavailability before it can be absorbed [14,15]. In terms of storage stability, freeze dried NanoCUR powder shows that the CUR inside PF micelle can be retained for a long time. Based on a study by Sahu et al. [12], CUR encapsulated with Pluronic F127 can be retained as much as 95.8% after 3 months, whereas encapsulation with Pluronic F68 retained 79.57% of CUR within the same period of time.

A plethora of studies have demonstrated the potential future applications of nanomaterials in various disciplines. Nanomaterials can be employed in modality treatment including targeted drug delivery systems [16], as well as integration into nano-scale probes for bio-imaging [17], attributed to its unique physiochemical properties, enhanced durability, and versatility. Recent advancements in technology coupled with researchers exploring nanomaterial applications in the health industry have led to new, innovative, and promising solutions to problems that conventional tools have yet to solve. However, nanomaterial research has also raised new concerns especially regarding its biocompatibility, which has prompted studies to assess the potential toxicity of nanomaterials. The use of nanomaterials in daily life causes an increase in the potential of the waste disposal of nanomaterials into the environment via manufacturers and users [18]. Nanomaterials have also been observed to bring damage to human health, thus making it difficult to administer it in vivo. Hence, the potential of nanotechnology in biomedicine is limited and concerning due to health and environmental factors.

The formulation and usage of NanoCUR in previous studies involving cancer cells [12,14] raised concerns regarding its toxicity effects toward normal healthy living organisms. NanoCUR has been shown to be able to modulate both the activity and expression of cytochrome P450 isozymes [13], which are responsible for approximately 75% of the total drug metabolism [19]. A study conducted using mouse embryos on the exposure of CUR showcased a significant toxicity effect, elucidating the generation of reactive oxygen species (ROS) and cellular apoptosis [20]. Another study using *Curcuma longa* extract to evaluate the embryotoxicity toward zebrafish embryos indicated a dose-dependent toxicity effect, where the toxicity effects were observed from the malformation, heartbeats, hatching rate, and mortality of the embryo [21]. Hence, to ensure the safety of a nanomaterial product, toxicity tests are essential to determine the possible toxic effect that the product may cause. Normally, experimental animals like rats will be employed, but in vivo experiments often warrant rigid and stringent animal ethics applications and specialized technical skills for animal handling. Furthermore, these experiments are expensive, time-consuming, and tedious, which could lead to inefficient data procurement [22]. Hence, it is imperative to have a viable alternative for the toxicity evaluation of nanomaterials.

The zebrafish (*Danio rerio*) is one of the leading in vivo model organisms used in many types of biological research due to its variety of unique features. They have small size, transparent embryos, high fecundity rate, rapid development [23], and ease of breeding, which could directly minimize the amount of space and cost needed for any experiments or toxicity testing [24]. Additionally, both human and zebrafish genes are homologous up to 70%; thus, their brain, muscle system, vascular system, digestive tract, and innate immune system are functionally and genetically similar [25,26]. All these features make zebrafish attractive as a model organism in studying toxicology with simplicity by assessing their developmental and morphological changes. Furthermore, zebrafish embryos have also been widely used, and have been considered highly effective in screening the toxicity of nanomaterials [27,28], as well as phytochemicals extracted from plants, such as *Piper sarmentosum* [29]. The salient features of the embryos, which are their greater sensitivity to undesired harmful effects and their transparent nature, make it easy for the embryos to be analyzed for the effects of toxicants on their organs, such as the heart, brain, liver, kidney, and others. Therefore, it allows direct observation of the periods of development and the assessment of the endpoints of toxicity. Testing using nanomaterials on zebrafish embryos also allows several parameters to be varied such as concentration, size, chemical composition, and time of exposure [27,28,30]. A previous study using zinc oxide nanoparticles on zebrafish embryos was not only able to assess embryo toxicity, but was also able to analyze the antioxidant defence system, ROS, and DNA damage [31]. Interference in the embryonic developmental stage might potentially lead to detrimental outcomes as it is an important phase in life. Research from a previous study has indicated that a treatment of CUR used on embryos was able to induce the generation of ROS that eventually leads to mortality [20]. Hence, the present study aims to investigate the biocompatibility of NanoCUR on the embryogenesis of zebrafish embryos, by evaluating the developmental changes (survival rate, hatching rate, and heart rate), morphological abnormalities (edema formation and scoliosis) and ROS generation, following exposure to different concentrations of NanoCUR, alongside native CUR, as well as PF as the only excipient, in order to obtain a more comprehensive toxicity profile of NanoCUR.

## 2. Results

### 2.1. Characterization of NanoCUR

**Figure 1:**
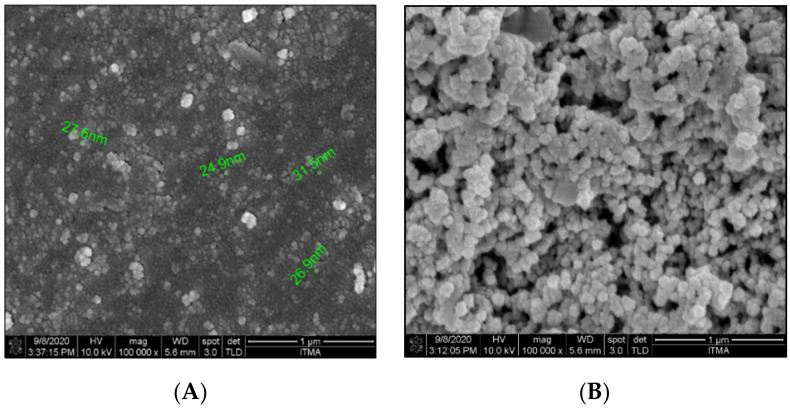
Particle morphology of NanoCUR; (**A**) with spherical and bead-like shape and the (**B**) size of NanoCUR particles measured by FESEM analysis that is comparable to the DLS results at 100,000× magnification. Scale bars represent 1 µm.

DLS was used to measure the hydrodynamic diameter, polydispersity index, and zeta potential. Analysis of data obtained through DLS enables the crucial assessment of nanoparticle behaviours in suspension and their tendency of agglomeration. Triplicate batches of NanoCUR produced were used for DLS analysis to evaluate batch-to-batch consistency. The DLS analysis of NanoCUR recorded a hydrodynamic diameter of 25.35 ± 0.46 nm, which was further corroborated by the FESEM analysis (Figure 1A). Polydispersity index (PDI) is a term used to describe the degree of non-uniformity in a sample. The value of polydispersity is influenced by the size distribution, agglomeration, or aggregation of the sample. NanoCUR recorded a narrow size distribution with a value of 0.21 ± 0.02, which indicates a monodispersed sample, with no formation of aggregates. The zeta potential registered by NanoCUR was −3.49 ± 1.10 mV, which was attributed to the non-ionic property of PF. NanoCUR was observed to be in self-assembled spherical and discrete shape with an average size ranged from 24 to 31 nm under FESEM analysis (Figure 1A). The NanoCUR samples were also found to be uniformly dispersed, which is in consensus with the DLS data (Figure 1B).

**Figure 2:**
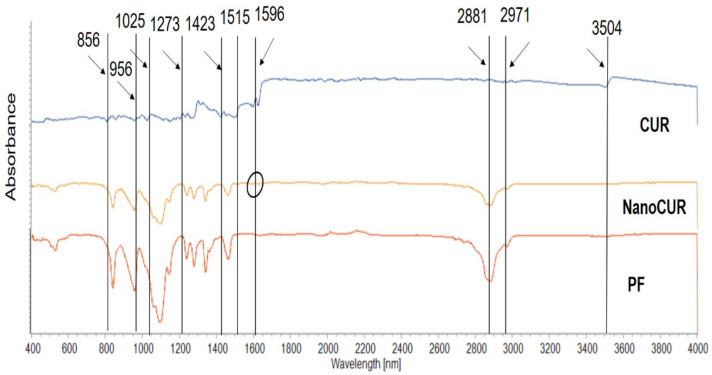
FTIR spectra of CUR, NanoCUR, and PF. The characteristic peaks of CUR, NanoCUR, and PF are shown. The FTIR profile of the NanoCUR samples indicates a new broad peak at around 1596 cm^−1^, which corresponds to the peak of CUR as indicated by the circled region. Loading of CUR into PF was also proven by the presence of a characteristic peak region at 3504 cm^−1^ and overlapping of other characteristic peaks of NanoCUR with that of PF.

The lyophilized NanoCUR, CUR, and PF were characterized using FTIR to identify the chemical bonds and functional groups present. The FTIR profile would be able to study the interaction between CUR in the PF structure by observing the characteristic peaks. Based on Figure 2, CUR has several main characteristic peaks, specifically at around 3504 cm^−1^ due to the presence of phenolic O-H stretching, a sharp peak at 1596 cm^−1^ due to the stretching vibration of the benzene ring, and at 1515 cm^−1^ representing C = O and C = C vibrations [32,33]. Other peaks were also recorded at 1423 cm^−1^ representing olefinic C-H bending vibration, 1273 cm^−1^ representing aromatic C-O stretching and 1025/856 cm^−1^ representing C-O-C stretching vibration. Meanwhile, PF shows several characteristic peaks around 2881 cm^−1^ and 2971 cm^−1^, representing the stretching of C-H from CH_3_ and CH_2_. Apart from that, ether bond and carbon–carbon double bond of the PF were observed around the 956 cm^−1^ and 1633 cm^−1^ spectral regions [14], which is in agreement with the molecular structure of PF, that consists of PPO and PEO unimers. The FTIR profile of the NanoCUR samples indicates a new broad peak at around 1589 cm^−1^, which corresponds to the peak of CUR at around 1596 cm^−1^. The loading of CUR into PF was also proved by the presence of a characteristic peak region at 3504 cm^−1^ and overlapping of other characteristic peaks of NanoCUR with that of PF. It is believed that the functional group belong to the peak of 1596 cm^−1^ shown in CUR and NanoCUR is the benzene ring of CUR, while the broad peak around 3504 cm^−1^ is attributed to phenolic O-H stretching vibration. Thus, the FTIR profile of NanoCUR was able to indicate a successful loading of CUR into the PPO hydrophobic core of PF, which shows the characteristic peaks of both PF and CUR.

**Figure 3:**
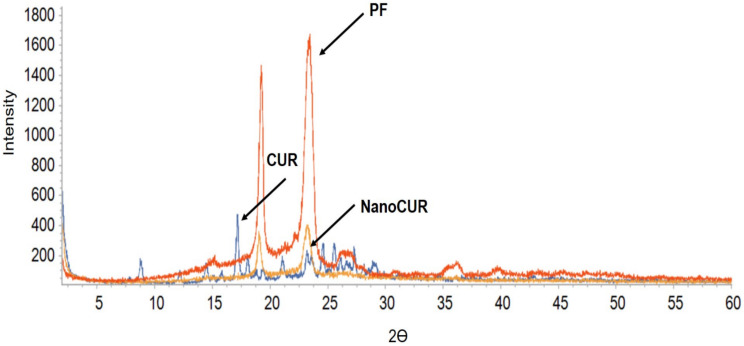
XRD patterns of native CUR and NanoCUR, along with PF as control, measured at 30 kV and 30 mA, with a scanning rate of 2°/min and 2ϴ angles ranging 2–60°. The XRD peaks in the CUR spectra indicate a crystalline nature. PF is also shown to be crystalline by the two distinctive peaks at 19.2 and 24. The XRD spectrum of NanoCUR shows a masking of the crystalline peaks of CUR upon encapsulation.

XRD data in Figure 3 shows three different spectra of CUR, NanoCUR, and PF. The graph pattern of CUR shows numerous peaks that indicates the crystalline structure of CUR, while PF was shown to have only two major characteristic peaks at 2θ of 19.2° and 23.4°. The peaks in PF remain in the XRD graph of NanoCUR, while various characteristic peaks of CUR could be barely noticed, which suggests that the NanoCUR surface structure is made up of PF. It also shows that CUR has been encapsulated in the micellar structure of PF, which masks the crystalline form of CUR.

The validated UV-Vis standard curve of CUR at different concentrations (0–10 µg/mL) (y = 0.1828x − 0.0272R² = 0.9994) in methanol was employed to quantify the drug loading (DL) and encapsulation efficiency (EE) of the NanoCUR. The drug loading was found to be 18.19 ± 2.02 mg CUR per 1 g of lyophilized NanoCUR powder with an EE of 82.14 ± 13.72%.

### 2.2. Survival Rate

**Figure 4:**
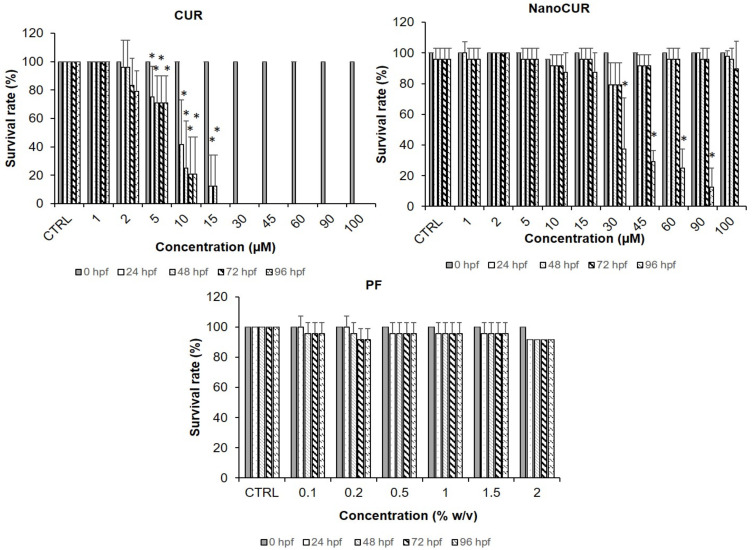
Survival rates (%) of zebrafish embryos treated with samples; CUR and NanoCUR at 1–100 µM, as well as PF at 0.1–2.0% *w*/*v*. Embryos exposed to embryo medium with and without 0.5% DMSO were used as control (CTRL). Data were averaged from three independent experiments and are shown as mean ± SD. * denotes significant difference to control, CTRL (two-way ANOVA, *p* ≤ 0.05).

Figure 4 shows the survival rate of zebrafish embryos upon being treated with the samples. Based on Figure 4, the embryonic survival rate was at 100% for the CTRL group and higher concentrations of PF at 1.0, 1.5, and 2.0% obtained survival rates higher than 80%. The survival rate for PF was consistent throughout the time of exposure from 24 h post fertilization (hpf) to 96 hpf, with no significant difference between time of exposure observed for the PF. As for the use of CUR treatments on the zebrafish embryos, the control treatment and 1 µM showed 100% survival rate. CUR at higher concentrations, starting from 10 µM, resulted in a significant decrease of survival rate, which further declined as the exposure time increased. Embryos did not survive the higher concentrations at 24 hpf which suggests that CUR possesses embryotoxicity toward the zebrafish embryo. The LC_50_ value of CUR was also calculated at 96 hpf and the value obtained was 6.302 ± 0.859 µM (Table 1). The survival rate of CUR-treated embryos exhibited a sharp decline at 24 hpf for concentrations above 10 µM. It was difficult to assess the time-dependent toxicity effect since concentration higher than 30 µM displayed 100% mortality rate at 24 hpf. However, it was observable for concentrations of 10 µM and 15 µM that they exhibited a time-dependent toxicity effect toward the zebrafish embryos. The survival rate displayed for 10 µM treatment of CUR was 41% at 24 hpf which further declined to 25% at 48 hpf, 20% at 72 hpf, and became consistent until 96 hpf. For the 15 µM treatment of CUR, the survival rate declined to 12.5% at 24 hpf continuing to 48 hpf and reached 0% at 72 hpf. Both results elucidated time-dependent toxicity of CUR toward the zebrafish embryos.

In contrast, for the NanoCUR treatment used on zebrafish embryos, all the concentrations of NanoCUR resulted in survival rates of above 70% from 24 hpf to 72 hpf. The survival rate of zebrafish embryos was not significant when compared to control for all concentrations and exposure from 24 hpf to 72 hpf. However, the survival rate of zebrafish embryo declined at 96 hpf in a concentration-dependent manner. A significant decline of survival rate was observed at concentrations of from 30 to 100 µM at 96 hpf. The LC_50_ value for NanoCUR was 28.4 ± 4.406 µM which was significantly higher compared to CUR (Table 1), which suggests that CUR exerts more toxicity compared to NanoCUR. As for the NanoCUR survival rate, the time-dependent and concentration-dependent toxicity effect was only observed at 96 hpf.

### 2.3. Hatching Rate

**Figure 5:**
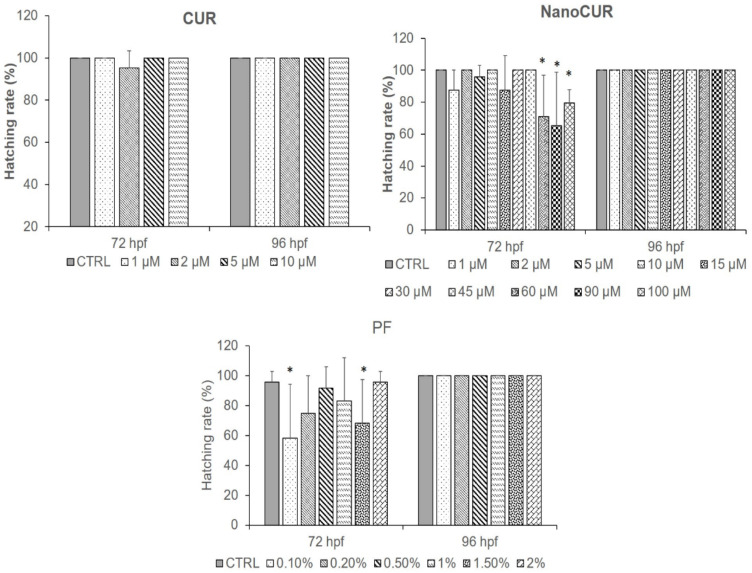
Hatching rate (%) of zebrafish embryos treated with samples, CUR at 1–10 µM, NanoCUR at 1–100 µM, and PF (0.1–2.0% *w*/*v*). Embryos exposed to embryo medium with and without 0.5% DMSO were used as control (CTRL). Data were averaged from three independent experiments and are shown as mean ± SD. Significant difference to CTRL is denoted by “*” (one-way ANOVA, *p* ≤ 0.05).

In this study, analysis of the embryonic hatching rate was carried out between 72 and 96 hpf. Figure 5 shows the hatching rates of zebrafish embryos exposed to CUR, NanoCUR, and PF. The hatching rate of CUR was only observed at lower concentrations (1–10 µM) due to the embryo toxicity of CUR at concentrations as low as 6.853 ± 1.079 at 48 hpf (Table 1), which resulted in no significant effect on the hatching rate when exposed to CUR. For the study in NanoCUR, a decline of hatching rate was also observed for high concentrations (60–100 µM) at 72 hpf. A decline of hatching rate below 70% was observed for PF for concentrations of 0.1% and 1.5% at 72 hpf. It is also important to note that there was no delayed hatching rate observed for any of the samples since at 96 hpf all the embryos had already hatched.

### 2.4. Heart Rate

**Figure 6:**
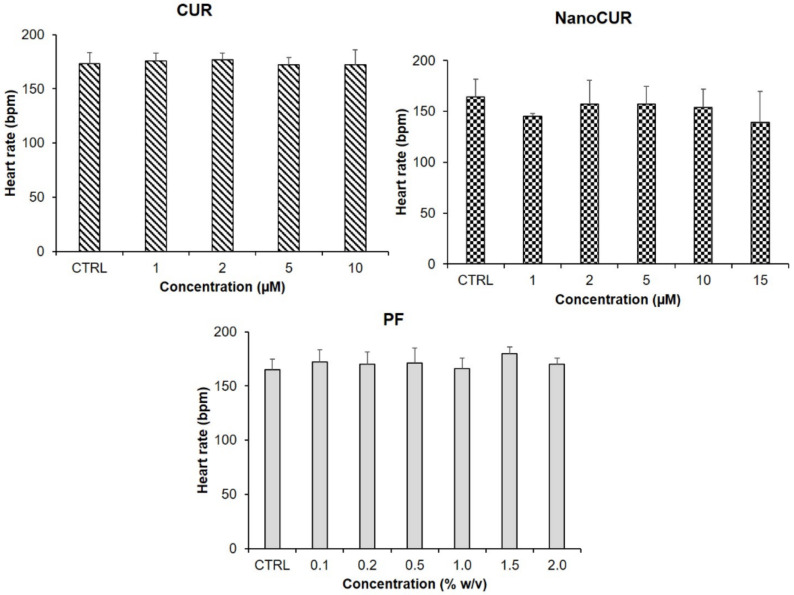
Heart rate (bpm) of zebrafish embryos treated with samples at 96 hpf, CUR at 1–10 µM, NanoCUR at 1–15 µM, and PF (1–2.0% *w*/*v*). Embryos exposed to embryo medium with and without 0.5% DMSO were used as control (CTRL). Data were averaged from three independent experiments and are shown as mean ± SD. No significant difference as compared to the control treatment (one-way ANOVA, *p* ≤ 0.05).

One of the crucial parameters to assess in the toxicity of toxicants in zebrafish embryos is the cardiac function, examined by methods such as measuring the heart rate. Figure 6 shows the heart rate of embryos exposed to CUR, NanoCUR, and PF, respectively. The heart rate of the zebrafish embryos was recorded at 96 hpf. During this hour, embryos had fully developed a visibly regular heartbeat and the normal embryonic heart rate of zebrafish embryos was in the range of 120–180 beats per minute (bpm). The result elucidated no significant decrease in the heart rate of zebrafish embryos at 96 hpf after being treated with CUR, NanoCUR, and PF. The heart rate observations for NanoCUR and CUR were conducted only at lower concentrations due to the mortality of the embryos exposed to higher concentrations at 96 hpf.

### 2.5. Morphological Assessments

**Figure 7:**
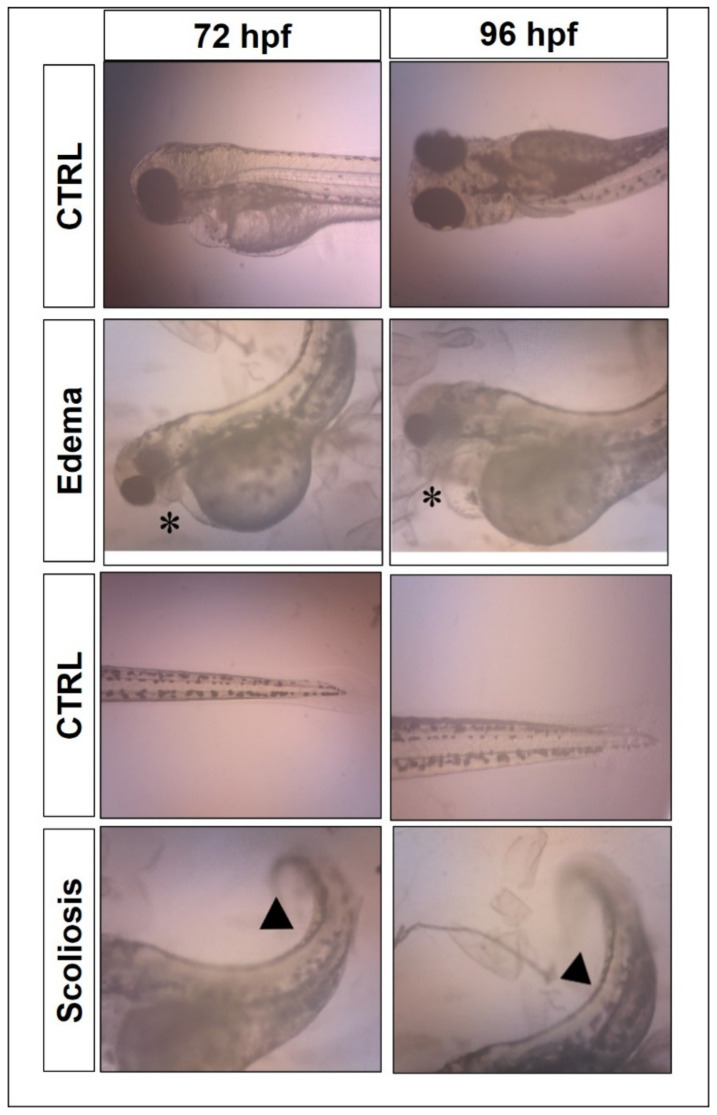
Images showing incidence of malformations in zebrafish embryo, including edema, marked by an asterisk (*) and scoliosis, indicated by a black triangle (▴), following exposure of CUR at 10 µM at 72 and 96 hpf. Embryos exposed to embryo medium with and without 0.5% DMSO were used as control (CTRL) and did not show any malformations at any time points.

Morphological assessment (Figure 7) was also conducted in the zebrafish toxicity assay to study the toxicity effect of the samples. The two common abnormalities seen in the present study were pericardial and yolk sac edema, as well as scoliosis malformations. PF and NanoCUR did not display any malformations up to 96 hpf for all concentration ranges. However, in CUR-treated embryos, malformation was detected at 10 µM, with apparent scoliosis and yolk sac edema formations at 72 hpf, which was further exacerbated at 96 hpf.

### 2.6. Reactive Oxygen Species (ROS) Assay

**Figure 8:**
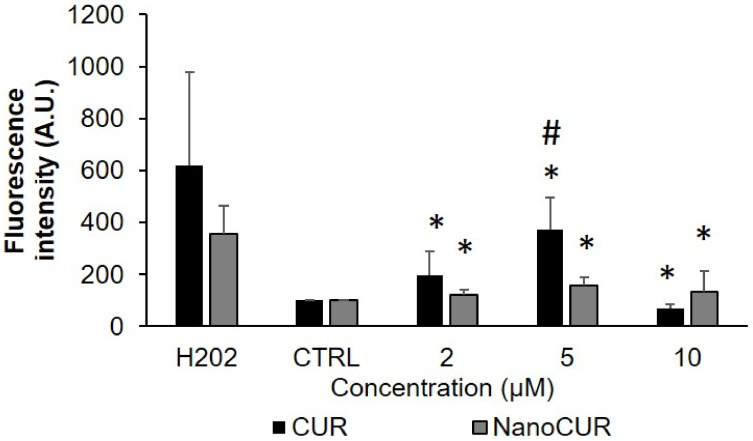
ROS generated following exposure of zebrafish embryos to CUR and NanoCUR at from 2 to 10 µM for 24 hpf as measured by fluorescence intensity (λex: 485 nm, λem: 530 nm). Embryos exposed to an embryo medium with and without 0.5% DMSO were used as control (CTRL). Data were averaged from three independent experiments and are shown as mean ± SD. * denotes significant difference to H_2_O_2_ and # denotes significant difference to CTRL (*p* ≤ 0.05).

ROS of the embryos treated with NanoCUR and CUR was measured upon exposure for 24 h at selected concentrations, 2, 5, and 10 µM, in which the treatment of both CUR and NanoCUR samples at these concentrations did not affect the survival of the zebrafish embryos deleteriously at 24 hpf. The generation of ROS was evaluated using DCFH-DA, where the fluorescence intensity was measured. In this study, H_2_O_2_ was used as a positive control to determine the ROS content. The intracellular ROS levels in the zebrafish embryos following exposure to CUR and NanoCUR were evaluated relative to the baseline ROS level in embryos incubated with an embryo medium (CTRL). Figure 8 shows the results obtained, where NanoCUR did not generate significant amounts of ROS compared to the CTRL. However, CUR treatment showed increasing generation of ROS from 2 µM to 5 µM and then declined at 10 µM.

## 3. Discussion

Curcumin (CUR), an active compound isolated from a common household spice, turmeric, has been researched for many years for its pharmaceutical properties, such as anti-inflammatory, antioxidant, and wound healing properties [34]. However, poor bioavailability, low aqueous solubility, instability at physiological pH, as well as its toxicity effect on normal cells have raised public concern and hindered further development of CUR in clinical settings. Due to these disadvantages of CUR, the effectiveness of CUR absorption is greatly reduced [9]. One study about the pharmacokinetic potential of orally administered CUR has shown that the plasma concentration of CUR was only about 50 mg/mL compared to the initial dose, 10–12 mg [35]. It shows that a significant amount of CUR was lost or metabolized long before it could reach the blood to perform its action. One of the methods to overcome the limitations imposed by native CUR, nanoformulation, has been vastly explored. In the present study, we elucidated the biocompatibility of a simple NanoCUR formulation, which has demonstrated biological properties in several previous studies [12,13,14] against the zebrafish embryonic model.

The size of nanoformulation is an important factor in determining the efficacy of a drug delivery system, along with its pharmacokinetics, biodistribution, and biological fate upon administration. The NanoCUR synthesized was found to be 25.35 ± 0.46 nm in size, which is suitable for both in vitro and in vivo experiments. Nanoformulations with particle size of above 200 nm or larger tend to activate the lymphatic system and are easily removed from blood circulation [36], while particles with sizes smaller than 10 nm are prone to be eliminated by renal filtration [37]. The zeta potential, which is a measurement of the surface charge covering the nanoparticle, was also measured in the present study that provides valuable insight on the stability of particles and on any possible interactions of the nanoparticles with biological systems. The zeta potential value should be low to minimize interferences with other biomolecules when administered, and nanoparticles with negative surface charge would assist in minimizing the cellular repulsion of nanoparticles [38]. Zeta potential of nanoparticles in the range of from +25 mV to −25 mV indicates a good degree of stability [39]. NanoCUR registered a low negative charge value, and is considered to be a good candidate for nanodrug applications.

The advancement of a pharmaceutical formulation depends highly on recognizing any potential toxicity issues and establishing its safety profiles, especially on formulations that use various types of excipients. The toxicity profile of NanoCUR and CUR samples was assessed in a zebrafish toxicity assay following characterization. As a vertebrate, zebrafish embryos are structurally and functionally similar to humans, and the contributions of genetics, anatomy, and physiology to embryonic nutrition can be explored [40]. It is strongly believed that the toxicity effects of NanoCUR in zebrafish embryos could contribute to a good correlation and reflection to human embryonic development. The toxicity profile was studied by observing the developmental changes and morphological abnormalities upon treatment of zebrafish embryos with samples. The parameters that were studied to assess the biocompatibility were survival rate, heart rate, and hatching rate. Moreover, the morphological abnormalities’ occurrence was also observed by monitoring the presence of edema, non-detachment of tail, lack of somite formation, and scoliosis. In the present study, the zebrafish embryos were treated with different concentrations (2–100 µM) of NanoCUR and CUR, whereas the concentration of PF used was 0.1–2% *w*/*v*, which is the concentration used to synthesize NanoCUR. The findings suggest that the concentration range for PF was not able to trigger any significant effects on the survival rate of zebrafish embryos. This is in agreement with a previous study by Hering et al. [41] which observed a triggered mortality effect of more than 50% for embryos treated with 10% *w*/*v* of PF, in comparison to embryos treated at a lower concentration, 1.25% *w*/*v*, that recorded a mortality rate below 10% at 72 hpf. Thus, it can be deduced that from the result obtained the concentration of 1% PF used during the synthesis of NanoCUR would not trigger the toxicity effect toward the zebrafish embryos in the present study, which is favourable for any nanoformulations with excipients. The results obtained from the present study suggest that CUR was toxic toward the embryos of zebrafish in a concentration-dependent manner based on the trend shown in Figure 5. In one study, zebrafish embryos were treated with CUR and its toxicity effects were observed, with an estimated LC_50_ value of the CUR recorded to be 7.5 µM at 24 hpf, and a concentration-dependent effect of CUR exposure was observed [42]. In contrast to CUR, NanoCUR displayed a delayed toxicity response where the toxicity effect was only significant at 96 hpf. Shamsi et al. [13] reported a similar finding in which the NanoCUR exhibited a delayed cytotoxicity effect toward HepG2 cells compared to the native CUR. It was deduced that the delayed response of NanoCUR was due to the slow and sustained release of CUR from the NanoCUR [12,14]. Furthermore, NanoCUR has slower rate of cell internalization due to the endocytosis system of the drug release compared to native CUR which has high membrane permeability due to its lipophilic properties [43,44]. These data also suggest that a prolonged exposure to NanoCUR may be necessary for the concentration of CUR from NanoCUR to reach its therapeutic threshold cellularly, which could then cause an increase in the toxicity of NanoCUR. Thus, it was observed that NanoCUR has an improved toxicity profile toward the zebrafish embryos compared to CUR.

The hatching rate of zebrafish embryos was not significantly affected by exposure to CUR at a lower concentration, which was in agreement with a previous study by Wu et al. [42] that only observed a decrease in hatching rate for embryos treated with CUR at concentrations exceeding 7.5 µM. Meanwhile, it was further observed that embryos exposed to NanoCUR at higher concentrations (60–100 µM) experienced a decline in hatching rate. As evidenced by the survival rate data, NanoCUR only exhibited toxicity at 96 hpf, with LC_50_ value of 28.4 ± 4.406 µM, whereas a decline in hatching was observed at higher concentrations (60–100 µM). This indicates that NanoCUR at higher concentration was able to accumulate in the embryos in a dose-dependent manner, hence penetrating the protective chorion of the zebrafish embryos and affecting the hatching rate. In contrast, PF has been recognized as a safe excipient, but in the present study, we could see that it was able to modulate the hatching rate in embryos at concentrations between 0.1 and 1.5% at 72 hpf. Previous researchers have established the ability of Pluronic to be a surfactant and a nanocarrier in drug delivery. The efficacy of Pluronic as a drug carrier has been demonstrated with doxorubicin prepared with a mixture of Pluronic^®^ L61 and F127 in Phase III of clinical trials with a reported ability to decrease the aggressiveness of tumor cells and deplete the stem cells of cancer [45]. A previous study has also demonstrated the use of Pluronic micelles as carriers for paclitaxel-lapatinib (PTX-LPT), with improved anti-tumor effects compared to native commercial drug Intaxel^®^ [46]. In addition, a study from Shamsi et al. [27] utilized PF as a surfactant to mitigate the toxicity of graphene oxide (GO) in zebrafish embryos. PF also possesses the thermo-reversible characteristic, which further enhanced the attention to its potential as a drug carrier for administration through oral, topical, and parenteral routes. As a surfactant, Pluronic^®^ can reduce water tension [47]. Previous studies have shown than non-ionic surfactants at high concentrations were able to impose negative effects on the growth and hatching rate of an aquatic organism, called fathead minnow, while simultaneously affecting the motility of fish and invertebrates [48,49]. Hence, further study is pivotal to identify the possible mechanism of PF that could have possibly brought about any detrimental changes in the embryonic environment, which resulted in the modulation of the hatching rate.

The morphological assessment was conducted to monitor any defects caused by the use of the treated samples on the zebrafish embryos. The assessed parameters in the morphological assessments were pericardial edema (PE), yolk sac edema (YSE), scoliosis (SC), non-detachment of tail, and lack of somite formation. Wu et al. [42] reported that zebrafish larvae treated with 5 µM of native CUR and higher showed developmental defects such as bent tail, edema at pericardial sac, retarded yolk, and short body length. Another study also reported that treatment of CUR at concentrations higher than 62.50 µg/mL displayed defects in body development such as kinked tail, bent trunk, body curvature, and yolk sac edema [21]. Embryos exposed to CUR at 10 µM at 72 hpf and 96 hpf exhibited malformations, which was evident from the incidence of yolk sac edema formation and scoliosis. However, the malformations’ incidence was not significant when compared to the respective control, due to the increased mortality and morbidity of embryos exposed to CUR for prolonged times. PF, at concentrations as high as 5%, has been reported to affect the morphology of embryonic chorion instantaneously upon exposure, which is postulated to be due to a leakage of the chorion fluid that reduces the pressure in embryos. It is worth noting that no morphological abnormalities were observed when zebrafish embryos were exposed to a lower concentration of PF (0–2% *w*/*v*) that was used for the formulation of NanoCUR. NanoCUR did not impose any significant malformations in treated embryos. Conversely, CUR displayed a time-dependent toxicity due to the weakening of the embryonic protective layer, which leads to a greater intake of CUR, and hence could influence the malformations observed at 10 µM [21].

Reactive oxygen species (ROS) has been reported to be one of the factors contributing to the toxicity of nanoparticles. The production of excessive ROS leads to the damage of proteins, DNA, and lipids compositions, leading to cell death. The generation of intracellular ROS can induce oxidative stress as well as cause damage to DNA and nucleic acids [50]. CUR at 5 µM generated a comparable ROS level to the negative control (0 µM), which is in congruent with a previous study that reported a significant ROS production in a mouse blastocyst model upon exposure to 24 µM of CUR, which further contributed this incidence to the mitochondrial-dependent apoptosis of the cells in the blastocyst [19]. CUR has also been shown to increase the accumulation of ROS in cells, which contributes to cell death in non-small lung cancer cells and MCF-7 human breast cancer cells at concentrations as low as 5.66 µg/mL [14] and 10 µM [49], respectively. As observed in Figure 4, CUR at a concentration of 5 µM demonstrated a significant decrease in survival rate as early as 24 hpf, which could be due to the intracellular generation of ROS. However, there was a significant increase of ROS production at between 2 and 5 µM of CUR, and the ROS value was seen to decrease for embryos treated with 10 µM of CUR, which could possibly be attributed to the decreasing survival rate of CUR (LC_50_ value for CUR at 24 hpf was reported to be 8.077 µM). Thus, the generation of ROS was not able to be accurately measured due to the mortality of the embryo at concentrations higher than the LC_50_ value recorded. Nevertheless, the findings suggest that CUR generated higher generation of ROS compared to NanoCUR, which was consistent with the toxicity results displayed upon the zebrafish embryos. Given that there are many other factors that could contribute to the toxicity of nanoparticles, further studies should consider looking into studying the effects of lipid peroxidation and enzymes. In addition, ROS could also be measured upon prolonged exposure to mimic the toxicity study and evaluate the capacity of NanoCUR to generate ROS, since the survival rate of the zebrafish embryos was only affected 96 hpf after exposure to NanoCUR.

## 4. Materials and Methods

### 4.1. Preparation and Characterization of NanoCUR

A thin film hydration method adapted from Shamsi et al. [13] was employed in the present study to prepare the CUR-loaded nanoformulation (NanoCUR). In this method, 0.1% *w*/*v* of CUR powder dissolved in methanol was mixed with 1% *w*/*v* of Pluronic dissolved in chloroform in a round-bottomed flask. The mixture then underwent dehydration using a rotary evaporator (Buchi R-200, Flawil, Switzerland) at 50 °C for 1 h. The resultant thin yellow film formed in the flask was left to dry under vacuum overnight at room temperature and was mixed with pre-warmed water (37 °C) for rehydration before centrifugation at 5000 rpm for 10 min (Beckman Coulter, Brea, CA, USA) to separate and remove unencapsulated CUR from the dispersion. Supernatant collected was filtered through a 0.22 µm membrane filter (Minisart^®^ Sartosium Stedim, Göttingen, Germany) to remove any larger particles. Finally, the final dispersion was characterized and freeze dried (VirTis benchtop K, Los Angeles, CA, USA) for 72 h at −55 °C. The freeze-dried powder was stored at room temperature in a sealed bottled, wrapped with aluminium foil to avoid exposure to moisture and light.

The data for the DLS analysis of NanoCUR were obtained using a Malvern Zetasizer Instrument (Malvern, PA, USA). In this study, NanoCUR samples were measured for their particle size, size distribution, and zeta potential in triplicates with the results shown as mean ± SD. Prepared NanoCUR samples were pipetted on the metal stub provided by the Institute of Nanoscience and Nanotechnology (ION2) and left to dry for 2 days. Air-dried samples were brought into the vacuum chamber of the FESEM (FEI Company, Hillsboro, OR, USA) and the morphology of nanoparticles was viewed with magnification of 100,000×. Samples of NanoCUR, CUR, and PF in powder were analyzed for their infra-red spectrum of absorption over a wide spectral range. The surface functional groups of NanoCUR, CUR, and PF were investigated with the FTIR technique using the Thermo Nicolet Model, Nicolet 6700 (Thermo Scientific, Waltham, MA, USA). Each disc was scanned at a resolution of 4 cm^−1^ over a frequency region of 400–4000 cm^−1^. Each spectrum represents an average of 32 scans. XRD patterns of all samples: CUR, NanoCUR, and PF were obtained at 30 kV and 30 mA, with a scanning rate of 2°/min and 2ϴ angles ranging from 2–60° with a XRD-600 Diffractometer (Philips, Amsterdam, The Netherlands) from the Department of Chemistry, Faculty of Science, UPM. The absorbance spectra of CUR and NanoCUR were recorded at full spectrum of visible light wavelength (200–900 nm) using an UV-Vis spectrophotometer (Jenway, Vernon Hills, IL, USA). CUR content was quantified based on the absorbance value at 425 nm using the same spectrophotometer. NanoCUR samples were prepared at 1 mg/mL in methanol and sonicated for 5 min to release the encapsulated CUR, and the dispersion was filtered (0.45 μm, PTFE membrane, Millipore, Burlington, MA, USA). The drug loading (DL) and encapsulation efficiency of CUR were determined by using Equations (1) and (2), respectively:

(1) $$\begin{array}{c} \mathrm{DL}=\frac{\mathrm{Weight}\;\mathrm{of}\;\mathrm{drug}\;\mathrm{in}\;\mathrm{nanoparticle}\;}{\mathrm{Weight}\;\mathrm{of}\;\mathrm{nanoparticle}} \end{array}$$

(2) $$\begin{array}{c} \mathrm{EE}=\frac{\mathrm{Drug}\;\mathrm{recovered}\;\mathrm{in}\;\mathrm{micelles}\;}{\mathrm{Original}\;\mathrm{drug}\;\mathrm{load}}\times \;100\% \end{array}$$

### 4.2. Toxicity Assessment in Zebrafish Embryos

A Danio Assay Kit Package was obtained from Danio Assay Laboratories Sdn. Bhd, Malaysia, equipped with zebrafish embryos, 96-well plates, Danio-embryo medium and manual instructions. The wild-type zebrafish (AB strain) was maintained by Danio Assay Laboratories according to standard in a recirculation system and under the permission of the Institutional Animal Care and Use Committee (IACUC), Universiti Putra Malaysia. The toxicity assay was conducted according to the protocol provided by the manufacturer. The embryos that were delivered would be less than 24 h post fertilization (hpf). The embryos were transferred into the petri dish provided, where the presence of dead embryos was checked on the top of a black surface. Embryos that were dead or coagulated were removed using a dropper and were excluded from further experiments, by observing the milky white, opaque, and dark appearances of the embryos upon arrival. Following this, the old embryo media was removed, and fresh embryo media was added to the petri dish. Healthy embryos were transferred into the 96-well plate (1 embryo/well) and incubated at 28 °C for a few hours for acclimatization prior to further experiments. The toxicity assay of zebrafish embryos was carried out using NanoCUR, CUR, and PF The embryos were arranged in three sets of 96-well plates which represented 3 different treatments (CUR, NanoCUR, and PF). Each concentration of treatment exposures consisted of 8 embryos, and was conducted in triplicate. Embryos exposed to the embryo medium with and without 0.5% DMSO served as the control (CTRL). All treatment solutions were refreshed daily [27]. The zebrafish embryos were treated with varying concentrations of the sample’s solution (1–100 μM for CUR in 0.5% DMSO and NanoCUR dissolved in water) and (0.1–2% of *w*/*v* of PF). The zebrafish embryos would then be observed at the interval of 24 hpf until 96 hpf. At each 24 h interval, the acute toxicity effects toward zebrafish embryos were recorded by observing the mortality, hatching rate, and heart rate. Each dead embryo was indicated as “1” and for the survived embryo, it was indicated as “0”. The heart rate of embryos was recorded at 96 hpf by counting the number of beats that occurred over a 15 sec-interval. This heartbeat was then extrapolated to 60 sec to provide average beats per minute [27]. In addition, to evaluate the significant impact of the treatments on the development of the embryos, the hatching rate of each embryo was also monitored at 72 and 96 hpf. The morphological abnormalities such as the presence of edema, scoliosis, non-detachment of tail, and lack of somite formation were recorded. The observation was carried out using an inverted microscope at 40x magnification (Nikon Eclipse TS-100). The embryos were also captured as images using the DinoLite microscope camera (Dino-Lite, Torrance, CA, USA) that was attached to an inverted microscope manually, with images analyzed using DinoCapture version 2.0 software (Dino-Lite, Torrance, CA, USA). The observation of all malformations was marked with number “1” which shows malformation presence, whereas “0” was marked to indicate a healthy embryo. The method was used for each type of the malformations mentioned above. The recorded data were tabulated in a table form for graphical data presentation.

### 4.3. Measurement of Reactive Oxygen Species

The reactive oxygen species (ROS) generated inside zebrafish larvae was measured using DCFH-DA [31]. Separate batches of zebrafish embryos were used to measure the ROS generation. The zebrafish embryos were treated with NanoCUR and CUR with concentrations of 2, 5, and 10 μM. NanoCUR was dispersed in water, while the CUR solution was prepared in 0.5% DMSO. Embryo medium with or without 0.5% DMSO was used as a negative control (CTRL), whereas 0.5 mM of hydrogen peroxide (H_2_O_2_) was used as positive control in this study About 10 embryos exposed to test samples for 24 h were pooled together and washed with cold PBS until no observable treated sample was traced. The embryos were then homogenized in cold PBS buffer and centrifuged at 12,000× *g* for 30 min at 4 °C (Sorvall Legend Micro 17R Microcentrifuge, Thermo Fisher Scientific, Rockford, IL, USA) where the supernatant was collected. The supernatant was transferred to a black 96-well plate (Corning, Corning, NY, USA) and incubated at room temperature for 5 min. After that, 100 μL of PBS was added with 8.3 μL DCFH-DA stock solution (dissolved in DMSO at 10 mg/mL) in each well. The 96-well plate was incubated at 37 ° C for 30 min in the dark. The fluorescent intensity was read by a microplate reader (Synergy H1, Biotek, Winooski, VT, USA) with an excitation wavelength of 485 nm and emission wavelength of 530 nm.

### 4.4. Statistical Analysis

Statistical analysis was conducted using Graph Pad version 8 (GraphPad Software Inc., San Diego, CA, USA) (2018). one-way or two-way ANOVA (analysis of variance) was used to determine the significance of toxicity effects for survival rate, heart rate, and hatching rate compared to the control group. A post-hoc test was also performed after ANOVA, such as the Dunnet post-hoc test, to determine the significance of exposure group value compared to control. Data were presented as the mean ± standard deviation (SD), from three or more independent experiments and the data were considered significant when *p* value was ≤0.05.

## 5. Conclusions

In summary, the findings suggest that NanoCUR could mitigate the toxicity of CUR, and hence can be considered as safe for drug delivery applications. PF did not elucidate any toxicity response in the present study with the concentrations used for preparation of NanoCUR, which is favorable since the focus of the study was to investigate the toxicity effect of CUR, loaded in a nanoparticle platform, with inert excipient. CUR exhibited a higher generation of ROS compared to NanoCUR, which was comparable to the toxicity response in the zebrafish toxicity assessment. Collectively, the data obtained in the present study could serve as the fundamental basis to pave a way for the development of NanoCUR as a more biocompatible alternative to CUR in clinical settings.

## Figures and Tables

**Table 1:** LC50 values of CUR, NanoCUR, and PF after exposure to zebrafish embryos for 24–96 hpf. The data are all presented as mean ± SD (*n* ≥ 3).

Exposure Time (hpf)	LC_50_ (µM)		
	CUR	NanoCUR	PF
**24**	8.077 ± 1.283	>100	>2% *w*/*v*
**48**	6.853 ± 1.079	>100	>2% *w*/*v*
**72**	6.379 ± 0.835	>100	>2% *w*/*v*
**96**	6.302 ± 0.859	28.4 ± 4.406 *	>2% *w*/*v*

* denotes significant difference to CUR at a specific time point (one-way ANOVA, *p* ≤ 0.05).

## Data Availability

Not applicable.

## Sample Availability

Samples of the compounds are not available from the authors.
